# Defending the Defender: Adversarial Learning Based Defending Strategy for Learning Based Security Methods in Cyber-Physical Systems (CPS)

**DOI:** 10.3390/s23125459

**Published:** 2023-06-09

**Authors:** Zakir Ahmad Sheikh, Yashwant Singh, Pradeep Kumar Singh, Paulo J. Sequeira Gonçalves

**Affiliations:** 1 Department of Computer Science and Information Technology, Central University of Jammu, Rahya Suchani, Bagla, Jammu 181143, India; zakirah786@gmail.com; 2 STME, Narsee Monjee Institute of Management Studies (NMIMS) Deemed to be University, Maharashtra 400056, India; 3 IDMEC, Polytechnic Institute of Castelo Branco, 6000-084 Castelo Branco, Portugal

**Keywords:** CPS security, cyber security, cyber attacks, adversarial attacks, poisonous attacks, evasion attacks, Generative Adversarial Networks

## Abstract

Cyber-Physical Systems (CPS) are prone to many security exploitations due to a greater attack surface being introduced by their cyber component by the nature of their remote accessibility or non-isolated capability. Security exploitations, on the other hand, rise in complexities, aiming for more powerful attacks and evasion from detections. The real-world applicability of CPS thus poses a question mark due to security infringements. Researchers have been developing new and robust techniques to enhance the security of these systems. Many techniques and security aspects are being considered to build robust security systems; these include attack prevention, attack detection, and attack mitigation as security development techniques with consideration of confidentiality, integrity, and availability as some of the important security aspects. In this paper, we have proposed machine learning-based intelligent attack detection strategies which have evolved as a result of failures in traditional signature-based techniques to detect zero-day attacks and attacks of a complex nature. Many researchers have evaluated the feasibility of learning models in the security domain and pointed out their capability to detect known as well as unknown attacks (zero-day attacks). However, these learning models are also vulnerable to adversarial attacks like poisoning attacks, evasion attacks, and exploration attacks. To make use of a robust-cum-intelligent security mechanism, we have proposed an adversarial learning-based defense strategy for the security of CPS to ensure CPS security and invoke resilience against adversarial attacks. We have evaluated the proposed strategy through the implementation of Random Forest (RF), Artificial Neural Network (ANN), and Long Short-Term Memory (LSTM) on the ToN_IoT Network dataset and an adversarial dataset generated through the Generative Adversarial Network (GAN) model.

## 1. Introduction

Cyber-Physical Systems (CPS) have gained a rise in applicability in many domains, including critical infrastructures, namely, the energy sector, manufacturing sector, dams, transportation, emergency services, etc. Their usage enhances efficiency and reduces human efforts by automating tasks. For instance, in power projects, CPS automates the process of power generation, transmission, and distribution. Previously, these systems were deployed in an isolated mechanism, wherein there was no remote accessibility mechanism available. Since the consideration of remote access to these systems via the Internet, there has also been a rise in the attack surface of these systems invoked by the redesigning of the system itself. Thus, connectivity to cyberspace makes these systems prone to many types of cyber-attacks [1]. Vulnerability exploitation of these systems could result in human and financial loss. Therefore, it is required to have some methodologies in place to ensure the detection and mitigation of these attacks [2]. Machine Learning (ML) and Deep Learning (DL) algorithms can learn the patterns of cyber-attacks both in online and offline modes and can possess the ability to detect complex attacks including zero-day attacks. Thus, their inclusion could be a better option for detecting cyber-attacks in CPS than the use of traditional signature-based mechanisms which fail to ensure performance against zero-day attacks.

**Figure 1:**
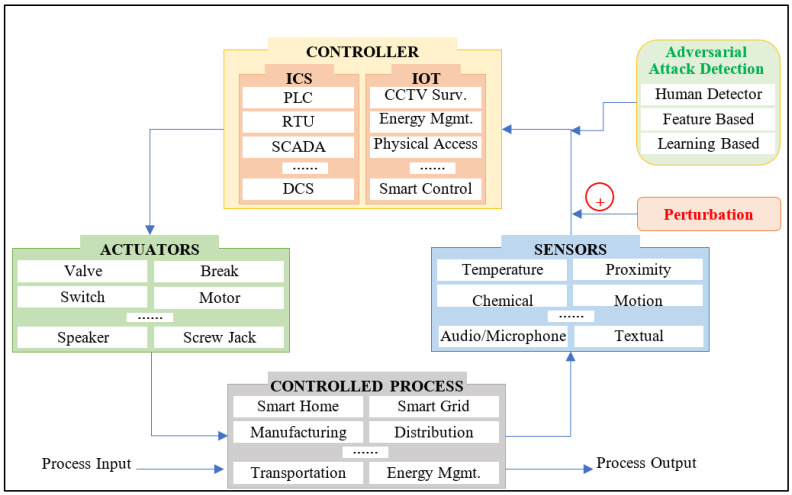
Adversarial attacks on the machine learning model in a CPS closed loop.

In order to consider the ML and DL-based mechanisms for cyber-attack detection in CPS, there is also a need to assess the security of these learning models. Specifically, it can be said that these learning models are also vulnerable to many types of adversarial attacks including poisonous attacks, evasion attacks, and exploratory attacks. Generally, the performance of these models depends on the training data, testing data, and the model itself, including its structure, so any sort of modification or deviation to the training data or testing data, or model structure, could result in malfunctioning of the model performance. For a general CPS, the data sources are surveillance sensors, textual data, or audio data, and the same is used to feed the learning model for training and testing purposes either in online or offline mode [3]. Modifications or perturbations to these data result in poisonous attacks and evasion attacks which occur at the training and testing phase of the model, respectively [4,5]. Model training is intended to learn the patterns of data and the testing is intended to evaluate the performance of the trained model on a similar set of patterns. Other than human intervention machines or external accessibility options, and repositories for storage, a CPS is generally composed of a closed-loop system containing the controlled process to automate [6], various sensors, controllers, and actuators, as shown in Figure 1 [7]. A controlled process can be any application area of CPS wherein the motive is to automate the process. For instance, in a hydro-power plant, the motive can be the automation of power generation, and for the same, various types of sensors can be used to measure different aspects, including a temperature sensor, pressure sensor, flow sensor, level sensor, and voltage sensor, to name a few. The sensor data are fed to the controller so as to invoke actuation as and when required. For instance, to stop the power generation, the controller can turn the valve off to stop the water flow towards the turbine. The data collected by various sensors can also be fed to an ML model to learn data patterns and take some intelligent decisions. The data flowing from sensors to the controller, though, can be perturbed by an adversary, which could result in performance degradation of the ML model [3]. In some simple scenarios, the human detectors or built-in mechanisms can notice the perturbations, but in complex scenarios, such as in the case of GAN-based perturbations [8], these mechanisms fail to detect the perturbations. Moreover, feature analysis-based and learning-based detectors can also be used as adversarial attack detection mechanisms. The other defensive mechanisms include data distortion, data decomposition-based methods on input data (i.e., data generated by sensors), and back-end mechanisms through structure and training enhancements [3].

In order to maintain an effective level of attack detection using ML and DL, there is a requirement to consider the security of these models as well. As these models could become victims of adversarial attacks, appropriate defensive measures should be in place to cope with any sort of performance degradation. As shown in Figure 1, an adversary can perform perturbation to the data collected from sensors, and the same data are fed to the detection module for training and testing. During the online model training phase, the model learns inaccurate patterns, whereas during the testing phase, the model evades detection and thus, results in performance degradation.

So, considering the aspects of CPS applicability, CPS security, the performance of learning models, and the security of learning models, the paper is intended to assess the performance of a learning model on a TON_IoT-based CPS dataset in an offline mode. Moreover, a Generative Adversarial Learning (GAN)-based model is used to create adversarial data samples similar to the original TON_IoT dataset with some tiny perturbations so as to evade detection. Once the model is trained on the original dataset, its performance is assessed on the testing samples of the original dataset and the adversarial dataset samples to assess the impact of GAN-based adversarial attack. In another phase, adversarial learning-based training is performed so as to prepare the model for adversarial samples.

### 1.1. Research Contributions

Machine learning (ML) and deep learning (DL) possess tremendous mechanisms to tackle known and unknown (zero-day) cyber breaches, but these learning models are also prone to various types of adversarial attacks at various phases. Considering the importance of these learning models and the associated risks, the following are the contributions of our paper:

i. Review of some important adversarial attacks on learning models and the preparation of a taxonomy thereof.
ii. Summary of significant methods of adversarial attacks and their attacking mechanism.
iii. Extending the use of a Generative Adversarial Networks (GAN)-based adversarial attacking mechanism for the cyber security domain. This includes a discussion of the generation of tabular adversarial datasets for cyber security, which are different from image and video datasets.
iv. Tabular adversarial data generation based on the TON_IoT Network dataset through the use of the GAN model.
v. Evaluation of the performance of learning models including Random Forest (RF), Artificial Neural Network (ANN), and Long Short-Term Memory (LSTM) against evasion and poisoning-based adversarial attacks.
vi. Proposing an adversarial learning-based security mechanism for Cyber-Physical Systems (CPS) and the evaluation of model performance under various scenarios.
vii. Generalizing the scalability and effectiveness of the proposed methodology by evaluating it on three learning models i.e., RF, ANN, and LSTM.
viii. Analyzing the computational requirements of the proposed methodology so as to assess its feasibility in constrained CPS networks.

### 1.2. Paper Organization

The paper is organized into various sections and the remainder of this paper is organized as follows. Section 2 discusses the adversarial consideration, including taxonomy, adversarial attacks, and adversarial defenses. Section 3 discusses the proposed methodology, which is followed by the Results and Discussion in Section 4. Finally, the paper concludes in Section 5 and indicates future directions. 

## 2. Adversarial Consideration

**Figure 2:**
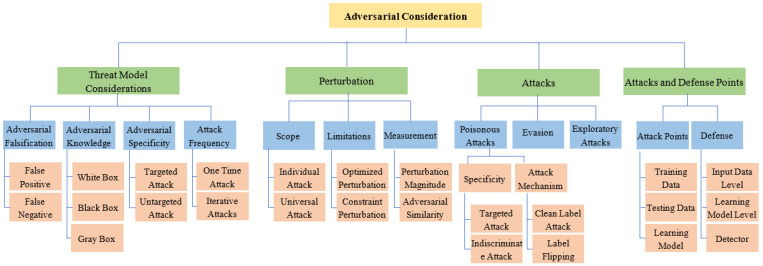
Various aspects of adversarial attacks and adversarial defense.

Machine learning and deep learning models possess the capability to ensure the security of Cyber-Physical Systems (CPS) [9]. This can be witnessed from the intelligent intrusion detection systems (IDS) based on various learning models including Naïve Bayes (NB), Decision Tree (DT), Support Vector Machine (SVM), Random Forest (RF), Deep Belief Network, Artificial Neural Network (ANN), and Long Short-Term Memory, to name a few [2,10,11]. Rosenberg et al. [12] in their work have analyzed that the learning models being utilized in the cyber security domain are also vulnerable to adversarial attacks. These learning models are also vulnerable to adversarial attacks, though, which are possible both at the training and testing phases and can arise because of the model itself or the dataset being fed to the model. Jadidi et al. [13] have assessed the security of machine learning-based anomaly detection in CPS and for the same, they have utilized Bot–IoT and Modbus IoT datasets. Moreover, they have generated adversarial samples through the Fast Gradient Sign Method (FGSM) and tested the effectiveness of the ANN-based learning model on original and adversarial datasets. Based on these adversarial impacts on learning-based security methods in CPS, we have prepared a taxonomy of adversarial consideration, as in Figure 2, depicting the threat model aspects, perturbation scope, perturbation measurement, types of attacks, and attack and defense points. The adversarial attacks have been primarily categorized into poisonous attacks, evasion attacks, and exploratory attacks. The poisonous attacks and evasion attacks are triggered at the training and testing phase, respectively, by perturbing or modifying their respective training and testing data subsets [4,14]. Considering the mechanisms of adversarial exploitation in machine learning, there can be specialized threat models to deal with adversarial threats. This may include the artifacts which are usually not considered in a general threat model, which include attacking frequency, adversarial knowledge about the learning model or training and testing data, adversarial specificity, and adversarial falsification, to name a few [3,4,15]. In addition, there can be perturbations of diversified scope and utilizing different perturbation measurement mechanisms. Data poisoning attacks are performed during the training phase which include the inclusion of adversarial data to the training dataset by performing perturbations like tiny perturbation or universal perturbation [16], utilizing Generative Adversarial Networks (GAN) for adversarial data generation [17], utilizing active learning-based approaches [18]. These attacks intend to either perform data perturbations to misclassify the input data samples or modify the output class itself to disturb the learning, which leads to performance degradation of the model.

In comparison to some related works, our proposed work is focused on developing a scalable approach to generate adversarial samples based on real-world CPS security datasets. Divergent to image and video datasets, CPS security datasets are usually in tabular form and require different methodologies to generate adversarial samples. Table 1 depicts the comparison of our proposed work to some related works. Most of the existing works only consider evasion-based adversarial attacks. In addition, among the compared works, only Jadidi et al. [13] have assessed the effectiveness of the adversarial learning approach and none of the works extend the use of Generative Adversarial Networks (GAN) for the generation of adversarial samples, which are significantly utilized for image- and video-based adversarial data generation. Moreover, none of the compared works have evaluated the computational time, which is a must-have aspect, as the CPS networks are generally constrained networks. To generalize the proposed methodology, we have also considered the evaluation of the effectiveness of our proposed methodology on three learning models: i.e., Random Forest (RF), Artificial Neural Network (ANN), and Long Short-Term Memory (LSTM). 

### 2.1. Adversarial Attacks

There are three distinct types of adversarial attacks: i.e., exploratory, evasion, and poisoning. In an exploratory attack, the attacker either changes the model itself or captures its learning parameters. Poisoning attacks are carried out during the training phase of the model whereas evasion attacks are carried out during the testing phase. A categorization of adversarial attacks has been depicted in Figure 2. The poisonous attack, also known as a causative attack, manipulates training samples so as to misclassify input data. Target attacks allow for the manipulation to be done in a way that misclassifies the input into the desired target class. On the other hand, a random attack incorrectly classifies input in any output class other than the original one [4].

There has been a significant rise in adversarial attacks on machine learning, and there are many discovered mechanisms to perform them. Some are based on perturbation such as tiny perturbation or universal perturbation [16], or are learning-based, such as the GAN-based [17], Active learning [18]. In addition, some adversarial attacking mechanisms rely on gradients such as the Fast Gradient Sign technique (FGSM), Momentum Iterative Fast Gradient Sign technique (MI-FGSM), Momentum-based [21], IGS (iterative Gradient Sign Method) [22], and HOUDINI [23]. Some other well-known adversarial attack mechanisms are the Jacobian-based Saliency Map Attack (JSMA) [24], the variant of Natural Evolution Strategies (NES) [25], ATNs (Adversarial Transformation Networks) [26], Deep Fool [27], ZOO (zero order) [28], One-Step Methods of a target class [29], ILCM (iterative least likely class method) [29], and Antagonistic Network for Generating Rogue Images (ANGRI) [30].

#### 2.1.1. Adversarial Attack Methods

Learning models are vulnerable to adversarial attacks due to modifications in data or model structure. The poisonous attacks happen as a result of training data modification intended to misclassify input data into a desired target class (targeted attack) or any class other than the original (indiscriminate attack or untargeted attack) [4]. This includes methods like clean label attacks [31] and label-flipping attacks [32]. The clean label attacks perform human-imperceptible perturbations to input features without flipping labels of corrupt input data, whereas the label-flipping attacks includes the change of labels of a fixed or constant fraction of the training dataset. Some authors call these poisonous attacks feature noise and label noise [5]. Shanthini et al. [5] demonstrated the impact of feature noise and label noise on three medical datasets and their evaluations showed that label noise causes a greater impact than feature noise. Zhang et al. [33] have evaluated the robustness of the Naïve Bayes (NB) classifier in a label-flipping-based poisonous attack scenario. They utilized the label-flipping attack by assuming that the attacker has limited knowledge about the classifier and can only manipulate dataset labels. For label flipping or noise addition, they used entropy_method and k-medoids. Aiming to achieve an enhanced False Negative Rate (FNR) under a label-flipping-based poisonous attack, they observed an increase of 20% FNR at the noise level of 20%. These evaluations prove the generalization “Naïve Bayes is robust to noise” mentioned by Gangavarapu et al. [34]. Label flipping can be performed through a random approach [33] or through other approaches that enhance misclassification. For instance, Biggio et al. [32] and Andrea et al. [35] proposed the heuristic utilization method, Han et al. [36] proposed Tikhonov regularization, Huang et al. [37] and Taheri et al. [38] proposed the correlated cluster method and Silhouette clustering based methods, respectively. A summary of some existing adversarial attack mechanisms is provided in Table 2.

#### 2.1.2. Adversarial Defenses

**Figure 3:**
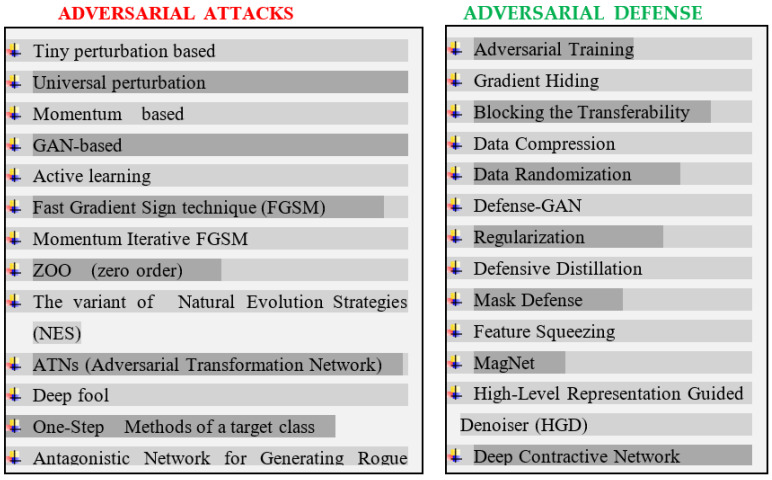
Adversarial attack and defense methods.

There are various defense mechanisms to deal with adversarial attacks. Some methods are based on the modification of data which includes Adversarial Training [40,44], Gradient Hiding [45], Data Compression [46], Blocking the Transferability [47], and Data Randomization [48]. Another category of adversarial defense mechanism alters the model itself to defend against adversaries. This model-based modification defense mechanism includes Defensive Distillation [49], Regularization [50], Feature Squeezing [51], Mask Defense [52], and Deep Contractive Network [53]. The third category of adversarial defense methods utilizes auxiliary tools to defend against adversaries. This category of defense includes MagNet [54], Defense-GAN [55], and High-Level Representation Guided Denoiser (HGD) [56]. Adversarial training or adversarial learning is a widely used approach in which the model is retrained so as to either correctly learn the classification of adversarial samples or create a separate class for adversarial samples. This method performs better defense in situations where all the possible adversarial samples are known [57]. For unforeseen adversarial samples, it has the least effectiveness. Various types of adversarial attack and defense methods have been depicted in Figure 3. 

## 3. Proposed Methodology

Our work is intended to generate an adversarial dataset using the GAN model and evaluate the performance degradation of the model on the same dataset. We initially train and test the model on an original dataset and then craft a similar dataset containing certain perturbations to misclassify the original input samples. To implement our methodology, we considered the existing ToN_IoT Network Dataset which contains 461,043 samples or tuples and 45 features [58,59]. Out of 45 features, 43 are input features and 2 are output features, namely, label and type. Among the 2 output features, we only used the type feature as we did not intend to evaluate multiple classifications but only the type of sample, either normal or attack. So, we considered 44 dataset features in total containing 43 input features and 1 output feature. With regard to the selection of learning models to evaluate the feasibility, scalability, and performance of our proposed methodology, we decided to consider multiple learning models. Based on the research of Rosenberg et al. [12], we analyzed that RF, ANN, and LSTM models have been widely used in the cyber security domain. The selected learning models have been implemented to evaluate their performance on the original ToN_IoT Network dataset and the GAN-based adversarial dataset. GAN-based adversarial attacks have been used in various works related to fake image generation [24,60], but there has been limited research related to the generation of a tabular dataset for the cyber security domain. One such example of fake image generation can be witnessed from the popular methodology “this person does not exist”, which generates fake images of people who do not exist in reality [61]. Hence, our research is intended to assess the feasibility of GAN-based adversarial attacks in the cyber security domain (relying on tabular datasets) and adversarial learning-based effectiveness against the adversarial attacks.

The performance of learning models has a dependency on the dataset and the model structures defined by their respective hyper-parameters. We have selected three learning models, i.e., RF, ANN, and LSTM, to evaluate the impacts of adversarial attacks and adversarial defense based on adversarial learning. The RF model has been defined with default hyper-parameters, except for the n_estimators, which is kept as 100. The ANN model is defined as a four dense-layered Sequential model with the number of neurons as 43, 24, 12, and 1 from input to output layer, respectively. The activation function relu has been used in the input and intermediate layers, whereas in the output layer, the sigmoid activation function has been used. Moreover, for optimization, the adam optimizer has been used. The LSTM model on the other hand is also a hardcoded model based on four layers of size 43, 50, 50, and 1 from input to output, respectively. Additionally, for optimization, the adam optimizer is used. 

Our proposed methodology mainly relies on CPS data and the sensor nodes in CPS are the main data sources for controlled processes. Intended to automate certain processes, CPS are controlled by controllers based on sensor data and the appropriate actions are triggered by the controller through actuators. Usually, the four (i.e., controlled process, sensors, controller, and actuators) components are the driving force in CPS, but the inclusion of an intelligent component between sensors and controller can enhance the security of the overall CPS through the utilization of the learning ability of the ML and DL model. The learning models trained on the CPS sensor data can learn normal and abnormal patterns during the training phase and the same learned capability can be utilized to check abnormalities in the CPS network. As learning models are also vulnerable to adversarial attacks, including poisoning attacks, and evasion attacks, we can also test the resilience of the proposed learning-based security methodology against adversarial attacks through the generation of adversarial data based on historical data patterns. To enhance the resilience of learning models against adversarial attacks, we can also make use of adversarial learning to train and test the model on adversarial patterns as well.

### 3.1. Problem Formulation

#### 3.1.1. GAN Attack

Let f(m,X,Y) be a model to be trained on a dataset or traffic flow X. The training aims to make the model learn the classification problem $X\overset{classify}{\to }Y$, where X is input data and Y is the output class or label. For a single sample or tuple, the model f(m,x,y) learns the classification of x sample to its target class y. On the other hand, adversaries aim to modify or generate adversarial samples $\tilde{X}$ through the evaluation of δ such that $\tilde{x}=x+\delta$ for each sample or tuple. The value of δ is calculated in such a manner so that the change in the original sample is undetectable to the human eye in the computer vision field or ensures the malicious behavior in the network security domain. Let a GAN model contain G and D as Generator and Discriminator model, respectively, Z be the random noise or latent space, G(Z) the adversarial data samples $\tilde{X}$ generated by Generator G, and G(z) be a single adversarial sample $\tilde{x}$ generated by G. Let $y_{0}$ be the label of the adversarial sample, and $y_{1}$ be the label of the original sample. The GAN model tries to generate an adversarial sample $\tilde{x}$ with label $y_{0}$ which is misclassified by D as $y_{1}$. If D classifies $\tilde{x}$ as $y_{0}$, the GAN invokes G to regenerate $\tilde{x}$. The process continues unless the D is not fooled to misclassify $\tilde{x}$ as an original sample with a label $y_{1}$. The adversarial dataset generated through GAN is fed to the learning model at the training phase (known as a poisonous attack) so as to impact the learning capability of the model. Moreover, in another scenario of assessing the impact of the learning model in an adversarial environment, a model trained on an original TON_IOT dataset is tested on an adversarial dataset (known as an evasion attack).

#### 3.1.2. Adversarial Learning

The adversarial learning strategy is used to learn the classification of adversarial samples $\tilde{X}$, wherein the adversarial dataset is split into training set ${\tilde{X}}_{Train}$ and testing set ${\tilde{X}}_{Test}$. In adversarial learning, the learning models are trained on the original TON_IOT training dataset $X_{Train}$ and adversarial training dataset ${\tilde{X}}_{Train}$. Moreover, the models are tested on the original testing dataset $X_{Test}$ and adversarial testing dataset ${\tilde{X}}_{Test}$. This evaluates the effectiveness of model learning in an adversarial environment.

### 3.2. Adversarial Dataset Generation

**Figure 4:**
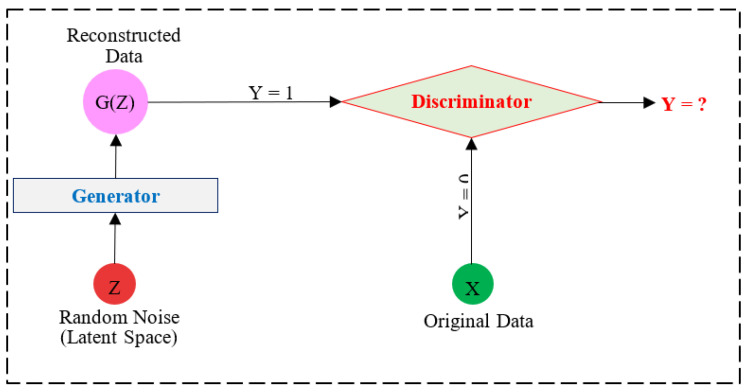
A general supervised GAN model structure.

A Generative Adversarial Network (GAN) is a deep learning-based model used to generate data. It is often used to generate data samples based on specific data entries. The GAN network has two core components, i.e., Generator (G) and Discriminator (D), as shown in Figure 4. Initially, the Generator is intended to generate a random data sample or tuple based on latent space or random noise. The generated data sample is fed to the Discriminator model which identifies the label Y of the data sample. If the Discriminator correctly identifies that the data sample is a generated sample, i.e., Y = 1, the sample is regenerated by the Generator model. The process continues until the Generator model fools the Discriminator model, i.e., label Y = 0 for each data sample generated.

**Figure 5:**
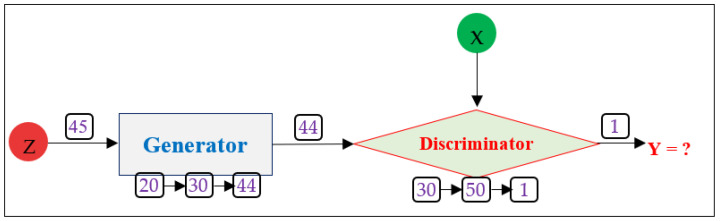
The structure of our GAN model for adversarial data generation.

As per the GAN structure, it requires two core models to perform adversarial data generation. These models are Generator (G) and Discriminator (D). Based on these facts, we implemented a GAN network with a Generator and Discriminator structure as shown in Figure 5. We implemented a four-layered Generator and Discriminator model. The structure of G is such that its output layer size is equal to the number of attributes in the original dataset and its input size is taken as 45, which is the attribute size of latent space. Overall, the structure of the Generator is 45→20→30→44, and the structure of the Discriminator is 44→30→50→1. The size of the Discriminator’s output layer is 1, which is because it has to represent only two values, i.e., Y = 0 (normal sample), and Y = 1 (attack sample). These two values of Y can be represented by a single neuron as well. The ‘relu’ is used as an activation function in all the layers of the Generator model except for the output layer that uses the ‘linear’ activation function. In the case of the Discriminator model, ‘relu’ is used as an activation function in all the layers except for the output layer, where ‘sigmoid’ is used as an activation function. Moreover, we used ‘adam’ as an optimizer in our GAN model. Overall, the structure of GAN (containing Generator and Discriminator) is hard coded in our case for adversarial data generation. We encourage readers to make use of hyper-parameter optimization (HPO) to define the structure of GAN, and other ML models.

To discuss the working mechanism of GAN, it takes input from latent space (Z) through its Generator which processes it to generate an adversarial sample representing characteristics of latent space. The generated samples are fed to the Discriminator model which also takes the original dataset (X) as its input to identify the label of the generated sample as per X. The aim of GAN is to make use of a Generator to generate an adversarial sample of X which is classified as an original/normal sample by the Discriminator model. If the Discriminator model classifies the generated sample as 1 (i.e., Y = 1) it means the generated adversarial samples are identified as adversarial, so the Discriminator model provides feedback to the Generator model to enhance the adversarial sample. The Generator model further modifies the previously generated sample and again, feeds it to the Discriminator. The process continues until the generated sample is classified as the original/normal data sample (i.e., Y = 0). The process intends that the GAN will aim to generate robust adversarial samples which ensure evasion from detection. Yet, it should be noted that the evasion from the Discriminator does not guarantee evasion from all ML and DL models. This needs to be assessed from the performance of ML and DL with such data samples. To generate an adversarial dataset using the GAN model, we utilized the latent space as input and generated 400,000 data samples in 100 epochs.

## 4. Results and Discussion

**Figure 6:**
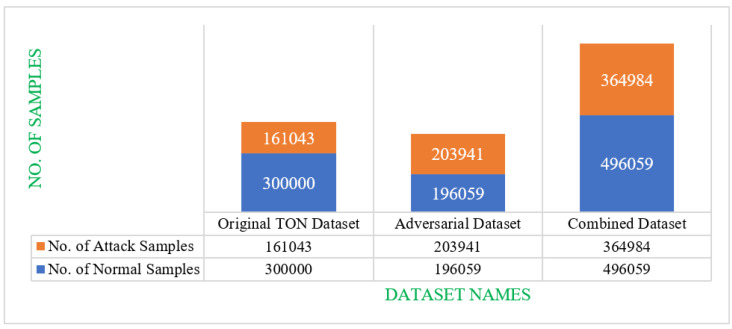
Statistics of the TON_IOT dataset, adversarial dataset, and combined dataset.

Our implantation is based on the performance assessment of learning models, namely, RF, ANN, and LSTM under different scenarios. Considering the vulnerability of learning models to adversarial attacks such as data poisonous attacks, and evasion attacks, we evaluate the performance of the selected learning models on an original TON_IOT network dataset [59] and GAN-generated adversarial dataset. The TON_IOT dataset has 45 features, and out of those, two features are output features describing the types of samples and attack names. The label feature indicates whether the samples are either attack (1) or normal (0), whereas the type mentions the exact attack name for the attack sample. In our study, we exclude the use of an attack name (i.e., type feature) as we do not consider the multiple classifications; rather, we only consider binary classification so as to check whether the sample is normal or an attack sample. So, out of 45 features, we consider 43 input features and 1 output feature. Initially, we perform dataset pre-processing of the original dataset to deal with empty cells and incompatible data types. Accordingly, we perform label encoding to deal with incompatible string datatypes and convert them to numeric data. In addition, we perform data normalization to ensure a common scaling of the whole dataset to speed up the training and testing process. Based on the TON_IOT Network dataset, we also generate an adversarial dataset using the GAN model. Moreover, we combine our adversarial dataset with the original TON_IOT Network dataset to assess the impacts of poisonous attacks, evasion attacks, and the capability of adversarial learning methodology to defend against the same. The statistics of all three datasets considered are depicted in Figure 6. For training and testing, we split the dataset in a 70:30 ratio for training and testing, respectively. We assess the performance of the selected learning models in four different scenarios, i.e., (a) train and test on the original dataset, (b) train on the original dataset and test on the original and adversarial dataset (evasion attack), (c) train on the original and adversarial dataset and test on the original dataset (data poisoning attack), and (d) train on the original and adversarial dataset, and test on the original and adversarial dataset (adversarial learning). All these four cases have been evaluated and discussed in the following sub-sections separately. The results of each of these cases are presented in Table 3.

### 4.1. Case 1: Performance on Original Dataset

**Figure 7:**
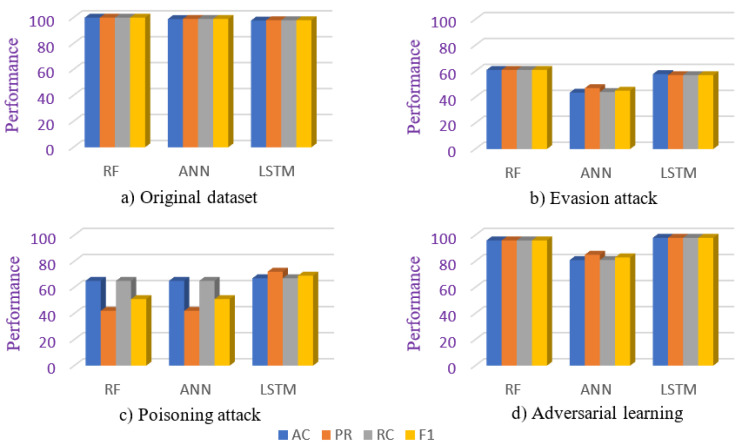
Performance achieved in different implementation scenarios.

To assess the performance of the selected learning models (i.e., RF, ANN, and LSTM) on the original ToN_IoT Network dataset, we split the dataset into training and testing sets in the ratio 70:30, respectively. Then, we defined each learning model and trained them on the training set of the ToN_IoT Network dataset. Once the models are trained completely, we assess their performance on the testing set of the ToN_IoT Network dataset. Based on our assessment, the RF and ANN achieved accuracy, precision, recall, and an f1 score of more than 99%, whereas the LSTM achieved accuracy, precision, recall, and an f1 score of 98%, as shown in Table 3 and Figure 7a. In terms of computational time requirements, RF took 32 s for training, and ANN took 18 min and 22 s for training. In the case of LSTM, we failed to execute the model several times on CPU-based system because of its computational requirements to retain the context in memory. This indicates that it is difficult to train the LSTM model on a real-time constrained CPS network. To assess the testing time requirements and the feasibility of the trained LSTM model to work on a constrained CPS network, we trained the model on a GPU-based system wherein the model took about 45 min for training and 8 s for testing. The testing time indicates that the trained LSTM model can be used in CPS networks as an intelligent intrusion detection system (IDS). In consideration of the complexity of the LSTM model, the LSTM model is trained on a GPU-based system in subsequent cases as well. 

### 4.2. Case 2: Evasion Attack

To assess the impact of evasion-based adversarial attacks during the testing phase, we train each of the selected learning models on the training set of the original TON_IOT Network dataset and test their performance on the testing set adversarial dataset. This evaluates the impact of the evasion-based adversarial attack on the model performance at the testing phase, and the generalization learning capability of the model. Based on our assessment, we observed performance degradation in all three models, as the RF, ANN, and LSTM only achieved an accuracy of 61%, 43%, and 57%, respectively, as shown in Figure 7b. Moreover, the precision, recall, and f1 score showed a big downshift in all three models, as shown in Table 3. In terms of timing requirements, the RF took 32 s for training, whereas the ANN model took 18 min and 22 s for training. In this case, also, the LSTM model could not be executed on a CPU-based system; hence, the model is trained on a GPU-based system to assess its feasibility of adoption as a trained model on constrained CPS systems as an IDS. On a GPU-based system, the model took 45 min 10 s for training, and 18 s for testing. The testing time indicates that the trained LSTM model can be used in constrained CPS networks.

### 4.3. Case 3: Data Poisoning Attack

This case evaluates the scenario of the learning models, wherein the models are trained on the training set of the original TON_IOT dataset and adversarial dataset and tested on the testing set of the original dataset. This replicates the scenario of a data poisoning attack, for instance, a model trained on a poisoned CPS dataset in offline mode and tested or deployed in online mode on a real-time CPS network. Specifically, in this case, we combine the adversarial dataset with the training set of the original dataset to assess the impact of the adversarial attack on the model performance. We shuffle the combined dataset and then split the same into training and testing sets in the ratio of 70:30, respectively. In the testing phase, we only utilize the testing set of the original dataset to assess the performance of the model. Based on our assessment, we observed that the selected models (i.e., RF, ANN, and LSTM) trained on the real/original dataset and poisoned dataset did not perfectly recognize the original data samples at the testing phase; these models result in an accuracy of 65%, 65%, and 67%, respectively. Moreover, there had been performance degradation in all three learning models in terms of precision, recall, and f1 score, as shown in Table 3 and Figure 7c. In terms of timing requirements, the RF took 8 min and 51 s for training, whereas the ANN model took 32 min and 23 s for training. In this case, also, the LSTM model is trained on a GPU-based system to assess the feasibility of trained LSTM on constrained CPS systems as an IDS. On a GPU-based system, the model took 58 min 16 s for training, and 15 s for testing. 

### 4.4. Case 4: Adversarial Learning

As we saw the performance degradation of RF, ANN, and LSTM models under GAN-based adversarial attacks, we utilized the adversarial learning-based strategy to enhance the model performances. For the same, we combined the original TON_IOT dataset and adversarial dataset and then split the combined dataset into a training and testing set in the ratio of 70:30, respectively. We trained each of the selected learning models on the training set of the combined dataset and tested them on the testing set of the combined dataset. Based on our evaluations, we observed enhancement in performance as RF, and LSTM achieved accuracy, precision, recall, and an f1 score of 96% and 98%, respectively. Moreover, the ANN achieved accuracy, precision, recall, and an f1 score of 80%, 85%, 81%, and 83%, respectively, as shown in Table 3 and Figure 7d. All three learning models have shown enhanced performance against evasion attack and poisoning attack through an adversarial learning approach. In terms of timing requirements, the RF took 8 min and 51 s for training, whereas the ANN model took 32 min and 23 s for training. For testing, RF and ANN took 7 s and 15 s, respectively. In this case, also, the LSTM model is trained on a GPU-based system to assess the feasibility of trained LSTM on constrained CPS systems as an IDS. On a GPU-based system, the model took 58 min 16 s for training and 22 s for testing. The training time indicates that it is difficult to train an LSTM model on a real-time constrained CPS network, whereas the testing time indicates that the trained LSTM model can be used as an intelligent IDS on constrained CPS networks.

From Figure 7a, we can analyze that all three selected learning models (i.e., RF, ANN, and LSTM) showed effectiveness on the original TON_IOT dataset with respect to accuracy, precision, recall, and f1 score. To analyze the impact of adversarial attacks during training (i.e., data poisoning attack) and testing phase (evasion attack), we generated a GAN-based dataset based on the lattice space of the original TON_IOT dataset. The impacts of evasion attack and poisoning attack on the selected learning models can be analyzed from Figure 7b and Figure 7c, respectively, which indicate the performance degradation of learning models in adversarial scenarios. Furthermore, to build resilient learning models for CPS security, we analyzed the importance of utilizing adversarial learning and the results for the same can be seen in Figure 7d. Comparatively, adversarial learning showed more effectiveness in terms of all four considered performance parameters than the adversarial scenario mentioned in Figure 7b,c.

**Figure 8:**
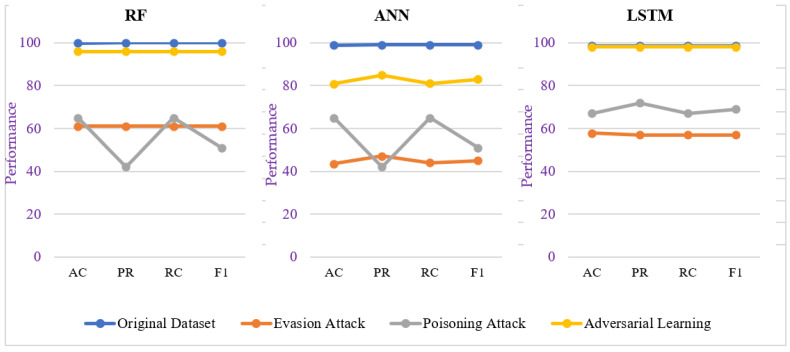
Performance comparison of selected learning models under normal dataflow, adversarial attacks, and adversarial learning scenarios.

From Table 3, we can infer that the GAN-based adversarial or data poisoning attack severely degrades the performance of a machine learning model. The actual impact of an adversarial attack can be observed in Cases 2 and 3 of Table 3. Out of the possible mechanism to deal with adversarial impact or build a robust machine learning model, we considered the adversarial learning-based strategy to learn the adversarial patterns. The use of adversarial learning enhances the performance of machine learning models against adversaries which can be observed from Case 4 of Table 3. The results of all three cases have also been visualized in Figure 8.

### 4.5. Discussion

The results reveal that the evasion and poisoning-based adversarial attacks severely degrade the performance of learning models in the CPS security domain as shown in Figure 8. Each of the selected learning models showed low performance against GAN-based adversarial attacks. The RF model has been more severely impacted by data poisoning attacks than evasion attacks, whereas the ANN and LSTM models have been more severely impacted by evasion attacks than data poisoning attacks. In an original TON_IOT dataset, all three selected learning models resulted in performances of more than 95% for accuracy, precision, recall, and f1 score, but in comparison to this baseline performance, the performance during the evasion attack and the data poisoning attack invoked big downshifts with regard to the same performance matrices. Out of the possible mechanism to deal with adversarial impacts or build robust learning models for the security of CPS, we considered the adversarial learning-based strategy to learn the adversarial patterns. The use of adversarial learning enhanced the performance of learning models against adversaries and the same can be witnessed in Figure 8. Through this approach, all three selected learning models performed better against adversarial attacks. More specifically, RF and LSTM achieved performances of 96% and 98%, respectively, whereas ANN achieved accuracy, precision, recall, and f1 score of 81%, 85%, 81%, and 83%, respectively. This indicates that the LSTM model performed much better than RF and ANN through an adversarial learning strategy and its performance against adversarial attacks was equivalent to its original baseline performance.

In terms of timing requirements, it can be analyzed from Table 3 that the RF and ANN can be easily trained and utilized as intelligent IDS on CPU-based networks; but as the LSTM model has greater complexity, it requires High-Performance Computing (HPC) for its training purpose. As we failed to train the model on a CPU-based system, we tried to assess the testing time requirements of the model, and for the same, we trained and tested the model on a GPU-based system. Post training and testing of the LSTM model on the GPU-based system, the results indicate that apart from training time requirements, the model requires time in seconds for testing purposes. Hence, we can conclude that the trained LSTM model can be adopted as an intelligent IDS in constrained CPS networks as well. 

## 5. Conclusions and Future Direction

Machine learning models have been widely adopted for the cyber security of CPS so as to ensure security against known and zero-day attacks. These models also possess the capability to deal with complex attacks and attacks of dynamic nature, but their performance depends on the level of training and training data. There exists a mechanism to deceive learning of these models at the training phase through the use of data poisoning-based adversarial attack. An attack at the testing phase only evades the real performance of the model, but an attack at the training phase degrades the learning capability of the model by feeding wrong patterns of input data (clean label attack) or output class (label flipping attack). Moreover, a model trained on correct data can also be deceived by an adversarial attack where the tiny perturbations invoke the model to misclassify data. 

Considering the importance of machine learning-based security for CPS, we thus proposed the use of an adversarial learning-based mechanism to train a machine learning model for the security of CPS. We have utilized a GAN-based adversarial attack mechanism as it utilizes a generator and discriminator modulus which ensures the evasion of perturbations for the adversarial data. We implemented RF, ANN, and LSTM models and evaluated their performance on the original TON_IOT Network dataset, evasion attack, data poisoning attack, and adversarial learning. On an original dataset, all three selected learning models performed at more than 95% in terms of accuracy, precision, recall, and f1 score, but they were severely impacted by evasion attack and poisoning attack. To make them robust against adversarial attacks, we utilized an adversarial learning-based approach, and through this approach, all three learning models resulted in enhanced performance. Out of all three selected models, the LSTM performed much better than RF and ANN and its performance against adversarial attacks was equivalent to its original baseline performance.

There are still certain challenges that need to be considered for researching similar kinds of problems. The adversarial learning method performs a better defense in situations where all the possible adversarial samples are known. For unforeseen adversarial samples, it has the least effectiveness. Moreover, attention should also be given to those types of adversarial attacks which utilize the mechanisms to attack the learning model itself rather than the training or testing data. We also encourage the readers to make use of hyper-parameter optimization to define the structure of GAN, and other learning models. 

## Figures and Tables

**Table 1:** Adversarial consideration for learning-based intrusion detection systems: comparison of aspects with some related works.

Ref.	Year	ML Model	Dataset	Adversarial Method	AML Taxonomy	Tabular Dataset	CPS Domain	GAN	Poisonous Attacks	Evasion Attacks	Adversarial Learning	Computational Time
**Jadidi et al.** [13]	2022	ANN	Bot–IoT, and Modbus IoT	FGSM	✗	✓	✓	✗	✗	✓	✓	✗
**Clements et al.** [19]	2021	KitNET (ensemble of AE and NN)	Real IoT Dataset, and Mirai	FGSM, JSMA, C&W, and ENM	✗	✓	✓	✗	✗	✓	✗	✗
**Qiu et al.** [20]	2021	Kitsune NIDS (AE-based)	Mirai, and Video Streaming	Gradient Based Saiency Map	✗	✓	✓	✗	✗	✓	✗	✗
**Proposed**	2023	RF, ANN, and LSTM	ToN_IoT Network	GAN	✓	✓	✓	✓	✓	✓	✓	✓

**LEGEND.** FGSM: Fast Gradient Sign Method, JSMA: Jacobian Base Saliency Map, C&W: Carlini and Wagner, ENM: Elastic Net Method, GAN: Generative Adversarial Network, AE: Auto Encoder, NN: Neural Network, AML: Adversarial Machine Learning, RF: Random Forest, ANN: Artificial Neural Network, LSTM: Long Short-Term Memory.

**Table 2:** Summary of existing adversarial attacking methods.

Adversarial Attack Method	Description	Equation/Methodology	Advantage	Disadvantage
Limited-memory BFGS (L-BFGS) [39]	To minimize the number of perturbations, the L-BFGS-based non-linear gradient-based numerical optimization method is used. It uses a box-constraint based optimization method.	$\begin{array}{l} \min x^{'}\\ =c\left|\left|n\right|\right|+J_{\theta }(x^{'},l^{'})s.t.x^{'}\\ \in [0,1] \end{array}$	Effectively generates adversarial samples.	It is a very computationally intensive, time-consuming, and impractical method.
Fast Gradient Sign Method (FGSM) [40]	Fast and simple gradient-based method for generating adversarial samples. It minimizes the maximum number of perturbations required to cause misclassification. Its mechanism is based on finding a small noise vector and the corresponding sign of elements of the gradient of the cost function.	$\tilde{x}=x+\in .sign\left(\nabla_{x}J\left(w,x,y\right)\right)$ where $\tilde{x}$ is an adversarial sample, ∈ is a noise vector, $\nabla_{x}$ is gradiant of x, and *J(w, x, y)* is the cost utilized to train the model with *w* as model parameters, *x* as model input, and *y* as model output.	Computationally efficient as compared to L-BFGS.	It adds perturbation to each feature.
Projected Gradient Descent (PGD) [41]	Unlike FGSM, which utilizes a one-step method for generating adversarial samples, the PGD is a multi-step variant of it.	$\begin{array}{l} x^{t+1}=\prod_{x+s}(x^{t}+\\ \propto .sign\left(\nabla_{x}J\left(w,x,y\right)\right)) \end{array}$	It invokes the strongest attack and is more powerful than FGSM.	Computationally more intensive than FGSM.
Jacobian-based Saliency Map Attack (JSMA) [24]	It uses feature selection to minimize the number of features to perform perturbation on. It uses saliency value in decreasing order to iteratively perform flat perturbation on features.	The Jacobian matrix of sample *x* is $\begin{array}{l} J_{F}\left(x\right)=\frac{\partial F(x)}{\partial x}\\ ={\left[\frac{\partial F_{j}(x)}{\partial x_{i}}\right]}_{i\times j} \end{array}$	Only a few features are perturbed.	Computationally more intensive than FGSM.
Deepfool Attack	It is an untargeted adversarial sample generation method. The method is based on minimizing the Euclidean distance between original samples and perturbed samples. It estimates decision boundaries between classes and iteratively adds perturbations.	---	Effective in generating adversarial samples with fewer perturbations and higher misclassification rate.	Computationally intensive than JSMA and FGSM. Moreover, it likely generates non-optimal adversarial samples.
Carlini & Wagner Attack (C&W) [42]	For adversarial sample generation, it utilizes L-BFGS-based optimization problems except for the usage of its box constraints and uses different objective functions.	$minimise\;D\left(x,x+\delta \right)\;such\;that\;C\left(x+\delta \right)=t\;C(x+\delta )\in {\left[0,1\right]}^{n}$ where *D* is the distance metric and is based on finding a minimum value *δ* which when added to the input sample *x* misclassifies to a new target class *t*.	Very effective in generating adversarial samples. This efficient method has defeated many state-of-the-art adversarial defense methods such as adversarial learning, defensive distillation, etc.	Computationally more intensive than FGSM, JSMA, and Deepfool.
Generative Adversarial Networks (GAN) [43]	Based on a two-player minimax game containing Generator G and Discriminator D. Generates adversarial attack data samples to bypass or deceive detection mechanisms.	$\mathrm{Min}-\max\;V\;(G,\;D)=\;E_{X\sim Pdata(x)}[\log\left(D\left(X\right))\right]+\;E_{z\sim Pz\left(z\right)}\left[\log\left(1-D\left(G(z\right)\right)\right)]$ where Ex: Expected value of overall data instances, Ez: expected value over all random inputs to the generator, Pdata (x): probability distribution of original data, Pz(z); distribution of the noise, D(X): discriminators estimate the probability of real data instances, and D(G(Z)): Discriminators estimate the probability of an adversarial data instance.	Generates adversarial/attack data similar to original data with the ability to evade defense mechanisms	Complexity and computational requirements of training the GAN model, and limitation of generating samples with little representative data.

**Table 3:** Results obtained by learning models under different scenarios.

Case	Case Name	Description	Training Phase		Testing Phase		Model	AC	PR	RC	F1	Training Time	Testing Time
			ODS	ADS	ODS	ADS							
**1**	Performance on Original Dataset	Performance evaluation on original TON IoT Network Dataset.	✓	✗	✓	✗	RF	99	100	100	100	32 s	2 s
							ANN	98	99	99	99	18 m 22 s	11 s
							LSTM	97	98	98	98	* 45 m 10 s	* 8 s
**2**	Evasion Attack	Evaluation of adversarial impact by testing the model on adversarial/generated dataset	✓	✗	✓	✓	RF	61	61	61	61	32 s	2 s
							ANN	43	47	44	45	18 m 22 s	21 s
							LSTM	57	57	57	57	* 45 m 10 s	* 18 s
**3**	Poisoning Attack	Performing data poisoning attack on training data	✓	✓	✓	✗	RF	65	42	65	51	8 m 51 s	2 s
							ANN	65	42	65	51	32 m 23 s	8 s
							LSTM	67	72	67	69	* 58 m 16 s	* 15 s
**4**	Adversarial Learning	Use of adversarial learning to enhance the model performance by learning the adversarial patterns.	✓	✓	✓	✓	RF	96	96	96	96	8 m 51 s	7 s
							ANN	80	85	81	83	32 m 23 s	15 s
							LSTM	98	98	98	98	* 58 m 16 s	* 22 s

**LEGEND:** PAttack: Poisoning Attack, EAttack: Evasion Attack, ODS: Original Dataset, ADS: Adversarial Dataset, AC: Accuracy, PR: Precision, RC: Recall, F1: F1-Score. * Executed on GPU-based server (failed to execute several times on CPU machine).

## Data Availability

Not applicable.
